# Dr. Salmon’s Pro Ective Virus Investigations

**Published:** 1884-04

**Authors:** 


Dr. Salmon's Protective Virus Investigations.—Dr. D. E. Salmon is entitled to much credit for being almost the only American investigator who has added to our knowledge of the methods of obtaining protective virus. In a contribution to the *Medical Record*, April 7, 1883, and in a letter of recent date, also in *Science* and the *Proceedings of the Kings County Medical Society*, Dr. Salmon has drawn attention to his special investigations. These have been made chiefly with the virus of chicken cholera. Following up the suggestive experiments of Chauveau with anthrax virus, Dr. Salmon used diluted virus, and injected it into fowls. After many trials he has devised an original method by which he obtains dilutions of a standard strength, and he claims with these to have obtained a vaccine that will be practically useful and much more easily made than that of Pasteur.

Dr. Salmon also claims to have shown that by injecting this very diluted virus, a local irritation and multiplication of germs is obtained, which confers immunity upon the whole organism. We congratulate Dr. Salmon upon his success in experimenting, but we can still doubt very much the practical utility of his vaccine. He himself has shown how variable is the susceptibility of different fowls to the poison, and it is very possible that even the most standard dilutions are not so fixed as is supposed. Chauveau and Peuch have not succeeded in doing more than experimentally demonstrating that the dilute vaccine may be protective.

We would suggest, as to Dr. Salmon's claim of showing the protective power of a local disturbance, that this has apparently been shown for years in the case of inoculations for pleuro-pneumonia. It is hard to prove, however, that the constitution is not slightly affected even in these cases.

